# Identify High-Quality Protein Structural Models by Enhanced *K*-Means

**DOI:** 10.1155/2017/7294519

**Published:** 2017-03-22

**Authors:** Hongjie Wu, Haiou Li, Min Jiang, Cheng Chen, Qiang Lv, Chuang Wu

**Affiliations:** ^1^School of Electronic and Information Engineering, Suzhou University of Science and Technology, Suzhou 215009, China; ^2^School of Computer Science and Technology, Soochow University, Suzhou 215006, China; ^3^The First Affiliated Hospital of Soochow University, Suzhou 215006, China

## Abstract

*Background.* One critical issue in protein three-dimensional structure prediction using either ab initio or comparative modeling involves identification of high-quality protein structural models from generated decoys. Currently, clustering algorithms are widely used to identify near-native models; however, their performance is dependent upon different conformational decoys, and, for some algorithms, the accuracy declines when the decoy population increases.* Results.* Here, we proposed two enhanced *K*-means clustering algorithms capable of robustly identifying high-quality protein structural models. The first one employs the clustering algorithm SPICKER to determine the initial centroids for basic *K*-means clustering (*SK*-means), whereas the other employs squared distance to optimize the initial centroids (*K*-means++). Our results showed that *SK*-means and *K*-means++ were more robust as compared with SPICKER alone, detecting 33 (59%) and 42 (75%) of 56 targets, respectively, with template modeling scores better than or equal to those of SPICKER.* Conclusions.* We observed that the classic *K*-means algorithm showed a similar performance to that of SPICKER, which is a widely used algorithm for protein-structure identification. Both *SK*-means and *K*-means++ demonstrated substantial improvements relative to results from SPICKER and classical *K*-means.

## 1. Background

A critical issue in protein three-dimensional (3D) structure prediction using either ab initio or comparative modeling involves identification of high-quality protein structural models from generated decoys [1–4]. According to the first principle of predicting protein folding, the native structure of the target sequence should be the conformation exhibiting minimal free energy [5]. According to this methodology, large-scale protein-candidate conformations are generated using ab initio or comparative methods [6–10]. Because accurate calculation of free energy remains unclear in theory [11–13], a protein-structure clustering algorithm is employed, and the structure located at the center of the largest cluster is considered the conformation exhibiting minimal free energy. In clustering algorithms, the 3D-structural similarity between two proteins is used as the distance metric. Currently, root mean square deviation (RMSD) and template modeling (TM)-scores [14] constitute the two most common metrics for determining 3D-structural similarity between candidates. Subsequent refinement steps are also performed based on the conformations detected by protein-structure clustering; however, the quality of the clustering algorithm directly affects the final results of protein prediction.

SPICKER is a simple, widely used, and efficient program used for identifying near-native folds. In this algorithm, clustering is performed in a one-step procedure using a shrunken, but representative, set of decoy conformations, with a pairwise RMSD cut-off determined by a self-adjusting iteration proposed by Zhang and Skolnick [15]. After benchmarking using a set of 1489 nonhomologous proteins representing all protein structures in the PDB ≥ 200 residues, Xu and Zhang [14] proposed a fast algorithm for population-based protein structural model analysis. Two new distance metrics, Dscore1 and Dscore2, based on the comparison of protein-distance matrices for describing the differences and similarities among models were developed [1]. Compared with existing methods using calculation times quadratic to the number of models, Dscore1-based clustering achieves linear-time complexity to obtain almost the same accuracy for near-native model selection.

Clusco [16] is a fast and easy-to-use program allowing high-throughput comparisons of protein models using different similarity measures (coordinate-based RMSD [cRMSD], distance-based RMSD [dRMSD], global distance test [GDT], total score [TS] [17], TM-score, MaxSub [18], and contact map overlap) to cluster the comparison results using standard methods, such as *K*-means clustering or hierarchical agglomerative clustering. The application was highly optimized and written in C/C++ and included code allowing for parallel execution, which resulted in a significant speed increase relative to similar clustering and scoring algorithms. Berenger et al. [19] proposed a fast method that works on large decoy sets and is implemented in a software package called Durandal, which is consistently faster than other software packages in performing rapid and accurate clustering. In some cases, Durandal outperforms the speed of approximate methods through the use of triangular inequalities to accelerate accurate clustering without compromising the distance function.

However, most of these methods are data sensitive, with both different protein targets and different modeling algorithms potentially resulting in large differences in detecting the center of clusters [20, 21]. One possible reason for this is that the free energy distribution varies greatly when using different decoy generated algorithms, such as those relying on ab initio and comparative modeling. Identifying the near-native conformation is also a memory and time-intensive task [22–24]. The *K*-means [25, 26] clustering algorithm is popular and has been successfully employed in many different scientific fields due to its robust performance in several previous applications [27, 28] and the relative simplicity of the algorithm. However, the efficacy of *K*-means clustering in protein-structure prediction has not been extensively studied.

In this paper, we proposed two enhanced *K*-means clustering algorithms to identify the near-native structures. The first one employs SPICKER to determine the initial centroids for basic *K*-means algorithm. Another one employs squared distance to optimize the initial centroids.

## 2. Methods

### 2.1. Data Sets of Benchmark

To comprehensively evaluate the methodology, we applied the algorithms to two representative datasets. The first dataset is I-TASSER SPICKER Set-II (http://zhanglab.ccmb.med.umich.edu/decoys/decoy2.html), which is widely used for evaluating the performance of protein decoys clustered algorithm [29, 30]. I-TASSER SPICKER Set-II contains the whole-set atomic structure decoys of 56 nonhomologous small proteins ranging from 47 residues to 118 residues, average with 80.88 residues. And the decoy average contains 439.20 conformations.

The second benchmark is CASP11 experimental targets which were generated by Zhang-Server and QUARK. We choose 12 hard and very hard targets from 64 CASP11 targets published on http://zhanglab.ccmb.med.umich.edu/decoys/casp11/. Hard and very hard targets indicate lower similarity of PDBs and more PDBs in the decoy. The targets without Zhang-Server and QUARK server results and with ZHANG-Server TM-score less than 0.6 are removed from the dataset. Each decoy contains around 1200–1500 conformations, average with 1520.83 conformations. These proteins ranged from 68 residues to 204 residues, average with 135.90 residues.

### 2.2. Classical *K*-Means Algorithm and 3D Distance Metrics

#### 2.2.1. Classical *K*-Means Algorithm

*K*-means algorithm is a typical clustering algorithm which is based on distance. It uses the Euclidean metric as the similarity measure. The closer the two objects, the greater the similarity *K*-means' important criterion. *K*-means considers that cluster is composed of many objects which are close in distance. Therefore, its final goal is to find out the compact and independent clusters. The selection of *k* initial clustering center has great influence on the clustering results, because in the first step *K*-means use a random selection of arbitrary *k* objects as the initial clustering center, representing an initial cluster. In each iteration, the remaining data set will be reassigned to the nearest cluster according to the distance. An iteration operation will be finished when all remaining data sets are assigned and new clustering centers will be calculated. When the new clustering centers are equal to the original clustering centers or less than a specified threshold, the algorithm will be finished. Euclidean metric is defined as follows:

(1) Euclidean  Metric=∑1Nxi2−xj2+yi2−yj2+zi2−zj2,

where *N* is the number of corresponding atoms between two objects *i* and *j*.

#### 2.2.2. Root Mean Square Deviation and Template Modeling Score

The similarity between two models is usually assessed by the root mean square deviation (RMSD) between equivalent atoms in the model and native structures after the optimal superimposition [31, 32].

RMSD alone is not sufficient for globally estimating the similarity between the two proteins, because the alignment coverage can be very different from approaches. A template with a 2 Å RMSD to native having 50% alignment coverage is not necessarily better for structure modeling than the one with an RMSD of 3 Å but having 80% alignment coverage. While the template aligned regions are better in the former because fewer residues are aligned, the resulting full-length model might be of poorer quality. Template Modeling Score (TM-score) function is a variation on the Levitt–Gerstein (LG) score [1, 33], which was first used for sequence independent structure alignments. TM-score is defined as follows:

(2) $$\begin{array}{c} TM\text{-}score=Max\left[\;\frac{1}{L_{n}}\overset{L_{a}}{\underset{i}{\sum }}\frac{1}{1+{\left(d_{i}/d_{0}\right)}^{2}}\;\right], \end{array}$$

where *L*_*n*_ is the length of the native structure, *L*_*a*_ is the length of the aligned residues to the template structure, *d*_*i*_ is the distance between the *i*th pair of aligned residues, and *d*_0_ is a scale to normalize the match difference. “Max” denotes the maximum value after optimal spatial superposition. RMSD, TM-score, and other metrics, such as GDT-TS (Global Distance Test) score and Qprob [34], can be used to evaluate the distance between the two structures. SPICKER enhanced the initial centers of the classical *K*-means algorithm.

One of the key limitations of the *K*-means algorithm concerns the positioning of initial cluster centers. As a heuristic algorithm, it will converge to the global optimum, with the results potentially dependent upon the initial cluster positions. In the classical *K*-means algorithm, the initial centers are randomly generated, and different initial positions consistently result in entirely different final cluster centers. SPICKER represents a simple and efficient strategy for identifying near-native folds by clustering protein structures generated during computer simulations. SPICKER performs this in a one-step procedure using a shrunken, but representative, set of decoy conformations, with the pairwise RMSD cut-off determined by self-adjusting iterations.

**Figure 1:**
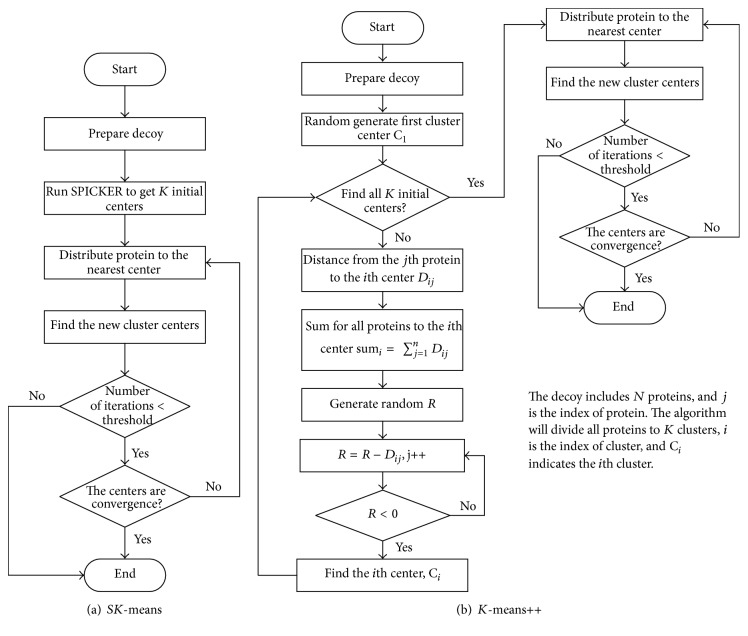
Algorithm flowcharts of *SK*-means and *K*-means++.

We proposed the first enhanced *K*-means algorithm, *SK*-means, which integrates SPICKER with *K*-means as Algorithm 1. In the 1st line Prepare_data() calculates the similarity of all proteins. In the 2nd line, startSpicker(*V*, *K*) executes the program, SPICKER, and gets *K* initial cluster centers. In the 6th line, function DistributeToCluster(**V**, C_*k*_, *n*) is to distribute the *n*th protein to the nearest cluster center C_*k*_ according to the distance matrix **V**. And in the 10th line, function CaculateNewCenter(C_**k**_) is to calculate the new center for current cluster C_**k**_. In the 19th line, Update() copies the new cluster center to the current cluster center. The flow chart of *SK*-means is depicted in Figure 1(a).

### 2.3. Initial Constraints Enhance the Classical *K*-Means Algorithm

Another enhanced *K*-means algorithm, *K*-means++ [35], was applied to detect the near-native conformation. The *K*-means++ algorithm maximizes the distance between initial cluster centers, which are not chosen uniformly at random from the data points that are being clustered. Each subsequent cluster center is chosen from the remaining data points, with probabilities proportional to its squared distance from the closest existing cluster center to that point. The flow chart of *SK*-means is depicted in Figure 1(b).

## 3. Results 

### 3.1. Benchmark on I-TASSER SPICKER Set-II

We compared the two enhanced *K*-means algorithms with SPICKER by randomly choosing the near-native conformation on I-TASSER SPICKER Set-II. The results are shown in Table 1 and demonstrated that the average TM-score of the first model detected by classical *K*-means was 0.5717, which was similar to results (0.5745) returned by SPICKER. Additionally, 33 (59%) of the 56 targets detected by *K*-means++ obtain TM-scores better than or equal to those of SPICKER, and 42 (75%) of the 56 targets detected by *SK*-means obtained TM-scores better than or equal to those of SPICKER. These results demonstrated that the performance of both of the two enhanced *K*-means algorithms outperformed SPICKER in situations involving larger populations of conformation decoys.

A statistical significance is important to indicate that the difference between two approaches' sample averages most likely reflects a “real” difference in the population. For practical purposes statistical significance suggests that the two larger populations from which we sample are “actually” different. *t*-Test (Student's test) is the most common form of statistical significance test. We implemented equal sample sizes *t*-test between four methods (*K*-means++, *SK*-means, *K*-means, and SPICKER) and random method in Supplemental Information Table  S1 in Supplementary Material available online at https://doi.org/10.1155/2017/7294519. Unfortunately, on I-TASSER Set-II dataset, none of the four methods show statistical significance with the random method as first row in Table S1. But when we only consider the data with decoy size less than 520 (2nd row in Table S1), *K*-means++ and *SK*-means showed more significant than *K*-means and SPICKER. These indicate that *SK*-means and *K*-means++ are more likely to be different with random method than *K*-means and SPICKER when the decoy size is less than 520.

### 3.2. Benchmark on CASP11 Hard Dataset

**Figure 2:**
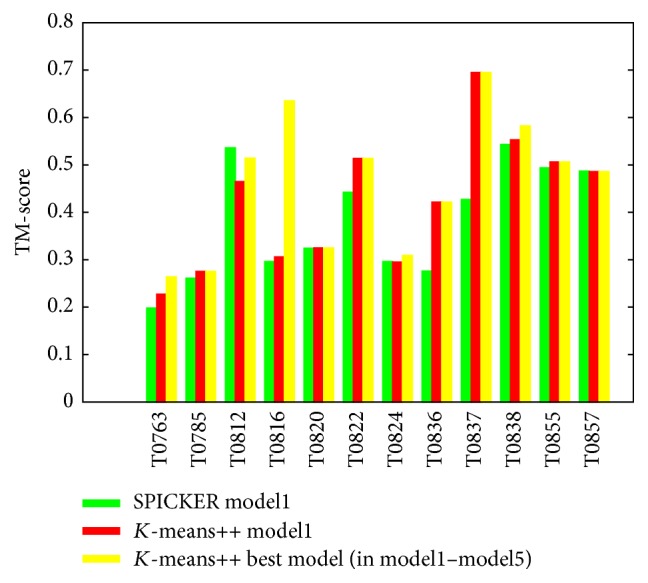
TM-score comparison between SPICKER and *K*-means++ on 12 CASP11 targets.

Figure 2 is a comparison of the TM-score between *K*-means++ and SPICKER. The green histograms are the TM-score of SPICER model1 from Zhanglab website (http://zhanglab.ccmb.med.umich.edu/decoys/casp11/). The red and yellow histograms are the TM-score values of model1 and the best model (in model1–model5) of *K*-means++, respectively. For all 12 CASP11 hard targets, 8 (66.7%) out of *K*-means++ model1 have higher TM-score than SPICKER model1. And on three targets (T0820, T0824, and T0857), *K*-means++ and SPICER have very similar results (TM-score difference is less than 0.01). *K*-means++ increase the average TM-score 10.5% from SPICKER's 0.38 to 0.42. *K*-means++ performances perfect on the target T0837 with TM-score 0.69 which is 60.5% higher than SPICKER's TM-score 0.43. 10 (83%) out 12 best models of *K*-means++ have higher TM-score than SPICKER. When comparing with *SK*-means, even though only 5 out of 12 model1s have higher TM-score than SPICKER, the average TM-score 0.38 of *SK*-means model1 is the same to SPICKER's. And the average TM-score 0.46 of *SK*-means best model, which is the same to average TM-score of *K*-means++ best model, is 28% higher than average TM-score 0.36 of SPICKER model1.

## 4. Discussion

### 4.1. Case Study on Three Targets

The two enhanced *k*-means methods got comparable results with SPICKER on most targets and demonstrated perfect advantages on some rich *α*-helix and *β*-stands targets. *α*-helix and *β*-stands are two most common secondary structure elements; they have been researched a lot and they are very important for protein 3D structure prediction. After exploring some targets, we find that *SK*-means possibly prefers to identify better *β*-strands targets and *K*-means++ possibly prefers to recognize better *α*-helix targets.

**Figure 3:**
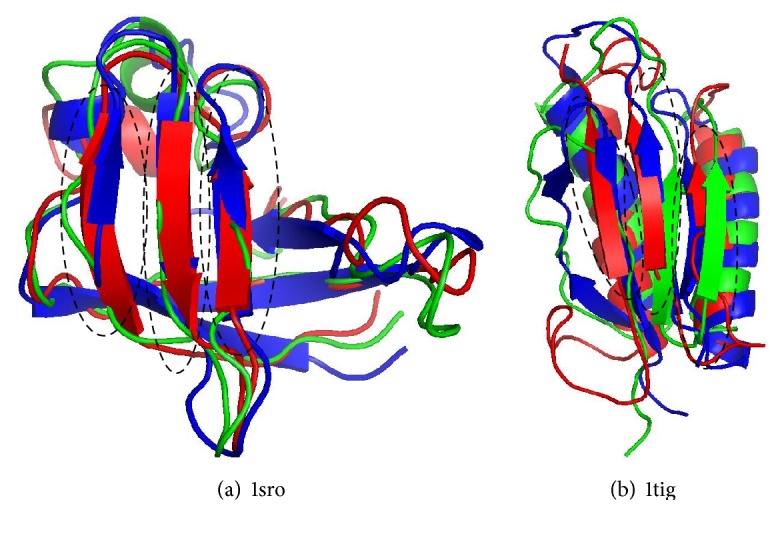
Superimposing of 3D structures of *SK*-means model1 (red), SPICKER model1 (green), and native (blue) on 1sro and 1tig. The black frames highlight the improvements of *SK*-means comparing with SPICKER.

The *SK*-means method achieved higher TM-score than SPICKER on some *β*-strands targets, such as 1af7, 1gpt, 1sro, and 1tig. We choose two targets (1sro and 1tig) and compared model1 which identified by *SK*-means (red) and SPICKER (green) with their native (blue) conformations in Figure 3. The black frames highlight the improvements of the *SK*-means algorithm relative to SPICKER results.  Figure 3(a) shows conformation identified by SPICKER (green) on 1sro; all three *β*-strands are shorter than those in the conformation identified by *SK*-means (red). For protein 1tig (Figure 3(b)), the conformation identified by *SK*-means (red), the three *β*-stand sections are closer to the native (blue) conformation than the structure identified by SPICKER (green). These results demonstrate that *SK*-means algorithm possibly can perform better on identifying *β*-stands.

**Figure 4:**
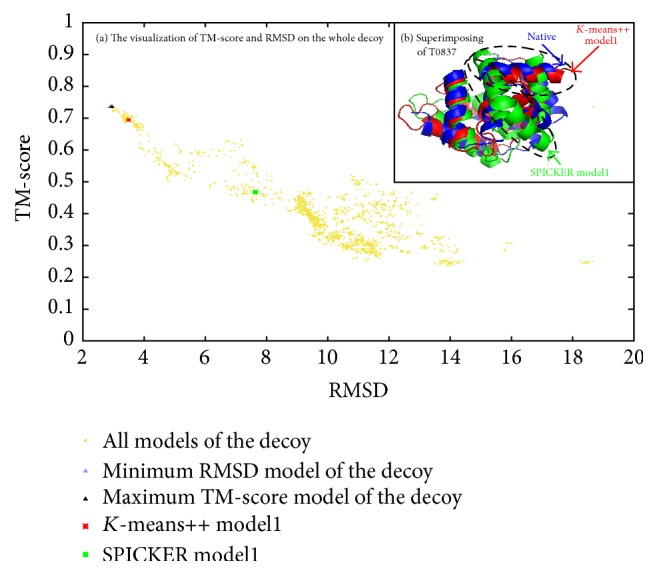
Visualized comparing in all models of the decoy and superimposing of 3D structures of T0837. (a) The visualization of TM-score and RMSD on the whole decoys. (b) The native structures, model1, identified by *K*-means++ and SPICKER are represented by blue, red, and green, respectively.

Figure 4(a) shows the distribution of TM-score and RMSD on the whole decoy with yellow points; points closing to the left-top are better. And we point out the minimum RMSD, the maximum TM-score, model1 identified by *K*-means++, and SPICKER with different point shape and color. In this figure, we find that model1 identified by *K*-means++ is closer to native than model1 of SPICKER on both measurement of TM-score and RMSD. In Figure 4(b), we find that T0837 is mainly consisted of *α*-helix. The conformation identified by *K*-means++ (red) is overlapped with the native conformation (blue) on most *α*-helix area. In the black frame, we mark an obvious difference between model1 structure identified by SPICKER (green) and the native structure (blue); the green *α*-helix has totally wrong direction. This probably validates our *K*-means++ algorithm having advantages in identifying better *α*-helix.

### 4.2. The Time and Space Complexity Analysis

Since, in classical *K*-means algorithm, every iteration requires calculation of the distance between each protein and each cluster center, the time complexity of classical *K*-means algorithm is *O*(*t∗K∗N*); here *t* is the number of iteration until cluster centers convergence.* K* is the specified number of clusters. And* N* is number of proteins in the decoy. The space complexity of classical *K*-means algorithm is *O*(*N* + *K*).

*SK*-means is combined by SPICKER and the classical *K*-means algorithm. The time complexity of SPICKER is *O*(*N*^2^*∗S* + *K∗N* + *S∗N* + *N*); *S* is the length of the protein. Therefore, the time complexity of *SK*-means is *O*(*N*^2^*∗S* + (1 + *K* + *S* + *t∗K*)*∗N*), the sum of *O*(*N*^2^*∗S* + *K∗N* + *S∗N* + *N*) and *O*(*t∗K∗N*). The space complexity of SPICKER is *O*(*N*^2^ + *N∗S* + *K∗S* + *S* + *N*). The space complexity of *SK*-means algorithm is largest of space complexity of SPICKER and classical *K*-means, *O*(*N*^2^ + *N∗S* + *K∗S* + *S* + *N*).

*K*-means++ is combined by initial centers choosing process and the classical *K*-means algorithm. The initial centers choosing process determines each center by the max distance to all other proteins, which has the time complexity *O*(*K∗N*) and the space complexity *O*(*N* + *K*). So the time complexity of *K*-means++ is *O*(*K∗N* + *t∗K∗N*). And the space complexity of *K*-means++ algorithm is *O*(*N* + *K*).

The time complexities of *SK*-means and *K*-means++ both have quadratic polynomial forms. The space complexity of *SK*-means and *K*-means++ has quadratic polynomial and linear forms, respectively.

## 5. Conclusions

Here, we developed two efficient methods for identifying high-quality protein structural models by enhanced *K*-means clustering algorithm (*SK*-means and *K*-means++). Based on the publicly available benchmark dataset (I-TASSER decoy set-II and), our results showed that *SK*-means and *K*-means++ were more robust than SPICKER at identifying conformational targets, with detection rates of 59% and 75%, respectively, exhibiting TM-scores better than or equal to those identified by SPICKER. Benchmarking on the CASP11 hard dataset, 8 (66.7%) out of 12 *K*-means++ model1 have higher TM-score than SPICKER model1. And the average TM-score 0.46 of *SK*-means best model, which is the same to average TM-score of *K*-means++ best model, is 28% higher than average TM-score 0.36 of SPICKER model1. These findings demonstrated that the two methods achieved better results at candidate-decoys populations conformations, possibly due to our improvements of initializing the cluster centers, thereby removing the element of randomness.

## Supplementary Material

Table S1: P-value Comparison between SK-means, K-means++, K-means and SPICKER with Random method in Statistical Significance Test.

## Figures and Tables

**Algorithm 1:**
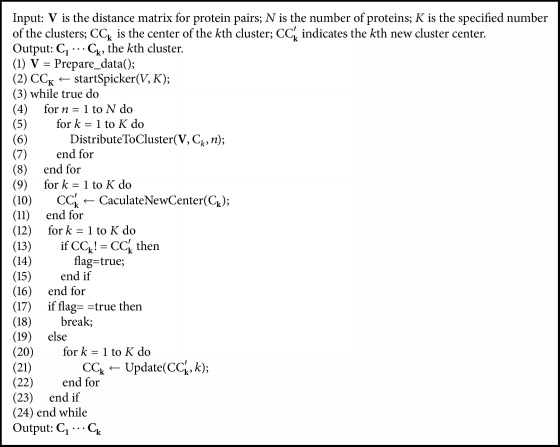
*SK*-means (**V**, *N*, *K*).

**Table 1:** Comparison between *SK*-means, *K*-means++, and SPICKER on 56 protein decoys.

Index	PDB	Len^a^	Size^b^	Best^c^	*K*-means++^d^	*SK*-means^e^	*K*-means^f^	SPICKER^g^	Random^h^
1	1abv	103	526	0.507	0.3701	**0.3834**	**0.4910**	0.3813	0.479
2	1af7	72	527	0.623	**0.5009**	**0.5009**	0.4820	0.4874	0.322
3	1ah9	63	510	0.696	**0.5040**	**0.4743**	**0.4740**	0.4657	0.434
4	1aoy	65	529	0.711	0.6482	0.6695	0.6695	0.6695	0.622
5	1b4bA	71	460	0.473	0.3815	0.4279	0.4270	0.4501	0.379
6	1b72A	49	534	0.697	**0.5397**	0.3917	**0.6410**	0.4923	0.562
7	1bm8	99	329	0.388	**0.4332**	**0.3787**	0.3320	0.3550	0.255
8	1bq9A	53	573	0.465	0.3540	0.3459	**0.3990**	0.3873	0.411
9	1cewI	108	452	0.748	**0.7294**	0.7154	**0.7290**	0.7187	0.617
10	1cqkA	101	284	0.885	0.8439	0.8539	0.8539	0.8539	0.815
11	1csp	67	315	0.753	0.7158	0.7158	0.7158	0.7158	0.686
12	1cy5A	92	273	0.893	0.8685	0.8839	0.8680	0.8839	0.876
13	1dcjA	73	525	0.368	**0.3299**	**0.3645**	0.3170	0.3264	0.334
14	1di2A	69	374	0.843	0.7622	0.7663	0.7620	0.7663	0.374
15	1dtjA	74	285	0.814	0.7901	0.7581	0.7370	0.7901	0.705
16	1egxA	115	352	0.827	0.7673	0.7673	0.7673	0.7673	0.768
17	1fadA	92	514	0.652	0.5716	0.5755	0.5755	0.5755	0.553
18	1fo5A	85	340	0.568	**0.5391**	**0.5391**	0.5230	0.5296	0.469
19	1g1cA	98	307	0.787	0.7473	0.7732	**0.7800**	0.7732	0.621
20	1gjxA	77	525	0.515	0.2375	0.3807	0.3810	0.4298	0.191
21	1gnuA	117	553	0.647	0.5353	0.5353	0.5350	0.5456	0.509
22	1gpt	47	469	0.553	**0.5130**	**0.5377**	**0.5060**	0.4927	0.517
23	1gyvA	117	337	0.776	0.7406	0.7406	**0.7540**	0.7406	0.753
24	1hbkA	89	300	0.708	0.6633	0.6633	0.6633	0.6633	0.599
25	1itpA	68	526	0.511	0.3069	**0.3152**	**0.3150**	0.3096	0.335
26	1jnuA	104	269	0.768	**0.7457**	0.7237	0.6980	0.7237	0.711
27	1kjs	74	548	0.5	0.3728	0.3728	0.3580	0.3728	0.313
28	1kviA	68	550	0.79	**0.7181**	0.6774	**0.7220**	0.6774	0.642
29	1mkyA3	81	285	0.552	0.4155	0.4155	0.4155	0.4155	0.384
30	1mla_2	70	335	0.775	**0.6742**	0.6226	0.6226	0.6226	0.609
31	1mn8A	84	545	0.457	0.2517	**0.3543**	**0.3540**	0.3285	0.310
32	1n0uA4	69	301	0.588	**0.4753**	**0.4746**	0.4524	0.4524	0.333
33	1ne3A	56	566	0.453	0.2523	**0.3943**	**0.3940**	0.3724	0.344
34	1no5A	93	426	0.419	0.3710	**0.4247**	**0.4240**	0.4054	0.500
35	1npsA	88	469	0.800	0.7671	0.7671	0.2810	0.7671	0.283
36	1o2fB	77	510	0.528	**0.3380**	**0.338**	**0.3370**	0.2690	0.379
37	1of9A	77	507	0.585	**0.5469**	0.494	**0.5460**	0.4940	0.554
38	1ogwA	72	520	0.890	0.7853	0.7853	0.7850	0.8622	0.78
39	1orgA	118	442	0.816	0.7440	0.7339	0.7440	0.7440	0.693
40	1pgx	59	562	0.551	**0.5824**	0.3216	**0.5160**	0.4446	0.51
41	1r69	61	291	0.824	0.7007	0.7255	0.7255	0.7255	0.827
42	1sfp	111	308	0.758	0.7453	0.7453	0.7454	0.7454	0.749
43	1shfA	59	536	0.836	**0.5649**	0.5070	**0.5640**	0.5070	0.408
44	1sro	71	515	0.648	**0.6513**	**0.6513**	0.5820	0.6158	0.583
45	1ten	87	294	0.851	0.8215	0.8215	0.7860	0.8215	0.781
46	1tfi	47	339	0.592	0.5061	0.5576	0.5520	0.5576	0.550
47	1thx	108	302	0.865	0.8000	0.8000	0.8000	0.8000	0.819
48	1tif	59	542	0.340	**0.3269**	0.2667	0.2660	0.3199	0.232
49	1tig	88	565	0.585	**0.5524**	**0.4596**	**0.4740**	0.4176	0.517
50	1vcc	76	551	0.455	0.3973	0.4066	0.3970	0.4066	0.291
51	256bA	106	506	0.814	**0.7657**	0.7578	**0.7650**	0.7578	0.723
52	2a0b	118	282	0.838	0.8083	0.8083	0.8083	0.8083	0.768
53	2cr7A	60	540	0.666	0.3589	0.5059	**0.5820**	0.5136	0.365
54	2f3nA	65	485	0.758	0.6403	**0.7322**	0.6510	0.7132	0.626
55	2pcy	99	435	0.637	0.6040	0.5795	**0.6460**	0.6233	0.527
56	2reb_2	60	550	0.403	**0.3902**	**0.378**	**0.3290**	0.3174	0.416

^a^The length of the protein sequence.

^b^The size of the models in the decoy.

^c^The best (maximum) TM-score of the models in the decoy.

^d^The TM-score of centroid model in the largest cluster selected by *K*-means++ (bold indicates better than SPICKER).

^e^The TM-score of centroid model in the largest cluster selected by *SK*-means (bold indicates better than SPICKER).

^f^The TM-score of centroid model in the largest cluster selected by *K*-means (bold indicates better than SPICKER).

^g^The TM-score of centroid model in the largest cluster selected by SPICKER.

^h^The TM-score of centroid model selected by random.

## Data Access

Programs and the data employed in the experiments are available at online http://eie.usts.edu.cn/whj/SK-means/index.html.
