# Supplementary material for: Identify High-Quality Protein Structural Models by Enhanced K-Means
Source: Biomed Res Int. 2017 Mar 22;2017:7294519. doi: 10.1155/2017/7294519 (PMC5381204; doi:10.1155/2017/7294519)
Supplement: Supplementary file 1 — Table S1: P-value Comparison between SK-means, K-means++, K-means and SPICKER with Random method in Statistical Significance Test. [file 7294519.f1.docx]

**Identify high quality protein structural models**

**by enhanced K-means**

Hongjie Wu^+^, Haiou Li, Min Jiang^+^, Cheng Chen, Qiang Lv ,Chuang Wu

**Supplemental Information**

**Table S1** P-value Comparison between SK-means, K-means++, K-means and SPICKER with Random method in Statistical Significance Test

| P-value | K-means++ | SK-means | K-means | SPICKER |
| --- | --- | --- | --- | --- |
| All data | 0.220 | 0.213 | 0.217 | 0.196 |
| Size<=520 | 0.171 | 0.159 | 0.382 | 0.187 |
